# Comparison of a reduced carbohydrate and reduced fat diet for LDL, HDL, and VLDL subclasses during 9-months of weight maintenance subsequent to weight loss

**DOI:** 10.1186/1476-511X-9-54

**Published:** 2010-06-01

**Authors:** James D LeCheminant, Bryan K Smith, Eric C Westman, Mary C Vernon, Joseph E Donnelly

**Affiliations:** 1 Brigham Young University, 269 SFH, Provo, UT 84606, USA; 2 The University of Kansas, 1301 Sunnyside Avenue, Lawrence, Kansas 66045, USA; 3 Department of Medicine, Duke University Medical Center, 4020 N Roxboro Street, Durham, North Carolina 27704, USA; 4 Private Practice, 21624 Midland Drive, Shawnee, Kansas 66218, Lawrence, Kansas, USA; 5 Center for Physical Activity and Weight Management, The University of Kansas, 1301 Sunnyside Avenue, Lawrence, Kansas 66045, USA

## Abstract

**Objectives:**

This study compared LDL, HDL, and VLDL subclasses in overweight or obese adults consuming either a reduced carbohydrate (RC) or reduced fat (RF) weight maintenance diet for 9 months following significant weight loss.

**Methods:**

Thirty-five (21 RC; 14 RF) overweight or obese middle-aged adults completed a 1-year weight management clinic. Participants met weekly for the first six months and bi-weekly thereafter. Meetings included instruction for diet, physical activity, and behavior change related to weight management. Additionally, participants followed a liquid very low-energy diet of ~2092 kJ per day for the first three months of the study. Subsequently, participants followed a dietary plan for nine months that targeted a reduced percentage of carbohydrate (~20%) or fat (~30%) intake and an energy intake level calculated to maintain weight loss. Lipid subclasses using NMR spectroscopy were analyzed prior to weight loss and at multiple intervals during weight maintenance.

**Results:**

Body weight change was not significantly different within or between groups during weight maintenance (*p *> 0.05). The RC group showed significant increases in mean LDL size, large LDL, total HDL, large and small HDL, mean VLDL size, and large VLDL during weight maintenance while the RF group showed increases in total HDL, large and small HDL, total VLDL, and large, medium, and small VLDL (*p *< 0.05). Group*time interactions were significant for large and medium VLDL (*p *> 0.05).

**Conclusion:**

Some individual lipid subclasses improved in both dietary groups. Large and medium VLDL subclasses increased to a greater extent across weight maintenance in the RF group.

## Introduction

Low fat diets have been shown to reduce some risk factors associated with coronary heart disease (CHD) and have traditionally been considered the standard of care for dietary treatment in overweight and obese adults [1]. Recently, a low carbohydrate diet has emerged as a potentially viable alternative diet to reduce some risk factors for CHD. Compared to a traditional low fat diet, low carbohydrate diets are associated with greater weight loss over six months, decreased triglycerides, and increased HDL cholesterol while other important risk factors such as, total cholesterol and LDL cholesterol tend to be reduced in similar fashion or reduction may be slightly greater on a low fat diet [2-7].

Subclasses for LDL cholesterol, HDL cholesterol, and VLDL cholesterol may provide a more complete cardiovascular risk profile than traditionally determined lipid values [8]. Recent investigations have reported subtle shifts in lipoprotein subclasses that tend to differ according to dietary carbohydrate and fat consumption [9-14]. As a result, there has been increased interest in how lipoprotein subclasses for LDL, HDL, and VLDL respond to manipulation of dietary carbohydrate and fat, particularly as it relates to CHD risk.

However, data comparing trends in lipoprotein subclasses according to carbohydrate and fat consumption are limited. In addition, existing studies comparing low carbohydrate and low fat diets have primarily reported lipoprotein subclasses during a period of weight loss and not weight stability [7]. The present study sought to improve upon these weaknesses in the literature. Therefore, the purpose of this study was to compare LDL, HDL, and VLDL subclasses in overweight/obese participants consuming either a reduced carbohydrate or reduced fat weight loss maintenance diet for nine months subsequent to three months of weight loss on a very low-energy diet (VLED). As there is currently no-agreed upon definition for a low carbohydrate diet [7] and the present study is not a very-low carbohydrate or ketogenic diet as reported elsewhere, the low carbohydrate and low fat diet groups will be described hereafter as reduced carbohydrate (RC) and reduced fat (RF) diet groups, respectively.

## Participants and Methods

### Participants

This study received approval from the University Human Subjects Committee and all participants signed an approved informed consent before beginning. Participation criteria and methodology for this study have been reported elsewhere [15]. In addition, participants in the present study were taken from a larger study, but only participants who had complete lipid subclass measurements were used in this analysis. Participants were overweight or obese (BMI >27 kg/m^2^) but otherwise healthy and free of disease, middle-aged adults, and previously sedentary. Participants were excluded if they smoked, were participating in special diets, unable to perform moderate-intensity physical activity, pregnant or lactating, or in active psychological or psychiatric counseling.

### Study Design

This study utilized a quasi-experimental design in which all participants were part of a behavioral weight management clinic and were prescribed a very low-energy diet (VLED) for three months followed by nine months in which participants consumed either a weight maintenance diet low in carbohydrate or low in fat. The rationale for this study design has been previously published [15].

### Intervention

#### Weight Management Clinics

Participants for this study were part of a larger clinical weight management program that included a dietary, physical activity, and behavioral component. The protocol and description for these clinical programs have been previously published [15,16]. In short, participants met weekly in a small group format (15-20 individuals) for the first six months and bi-weekly during the final six months. The meeting protocol included: initial weigh-in for body weight, discussion of self-reported data for diet (liquid shakes, carbohydrate grams, or fat grams) and physical activity (minutes and pedometer counts), and instruction for diet, physical activity, or behavioral change strategies associated with weight management. As participants met separately by dietary group assignment, participants received diet-specific information and training during the weight maintenance period; otherwise, clinic meetings were identical between dietary groups.

#### Very Low-Energy Diet

During weight loss (months 1-3), a liquid VLED was utilized. Liquid supplements (Health Management Resources, Boston, MA) were taken at five different intervals daily totaling ~2092 kJ/day. A vitamin and mineral supplement was taken twice per day along with the liquid supplements. As this was a weight-loss maintenance study, a minimum weight loss of 10% of initial body weight was required during VLED to progress to the weight maintenance stage (months 4-12). Participants reported their weekly liquid supplement total at each group meeting. In addition, the VLED period was medically managed by the study physician during its entire duration.

#### Weight Maintenance Diets

In order to decrease the likelihood of adverse events, participants progressively transitioned from liquid to solid foods, that were diet-specific (reduced carbohydrate or reduced fat), during the fourth month. By the end of month four, participants were solely consuming solid foods according to dietary group assignment and continued in this manner for the duration of weight maintenance. The weight maintenance energy level for each participant was determined utilizing the Harris-Benedict equation for resting metabolic rate and adjusted for physical activity using a factor of 1.4 [17]. In conjunction with the weight maintenance energy intake level, the RC diet participants were assigned ~20% of their energy to be consumed from dietary carbohydrate and for the RF diet participants were assigned ~30% of their energy to be consumed from dietary fat. To facilitate dietary tracking, each participant received an upper-limit daily gram level for either carbohydrate or fat consumption and recorded their gram consumption each day. Each participant reported their weekly carbohydrate or fat gram intake at the group meeting. In addition, gram intake was reported via email, phone, or fax if a meeting was missed and during the off-week when meetings were bi-weekly.

### Assessments

#### Anthropometrics

At baseline and prior to each group meeting, body weight was assessed using a digital scale (Befour, Inc., Saukville, WI) accurate to ± 0.1 kg. Participants were weighed in street clothes and without wearing shoes. Height was measured using a stadiometer (Perspective Enterprises, Portage, MI). Waist circumference was assessed at the narrowest portion of the waist between the lowest rib and iliac crest using a spring-loaded tape measure (Creative Health Products, Ann Arbor, MI) at baseline and throughout maintenance.

#### Energy Intake

Energy, carbohydrate, fat, and protein intake were assessed via 3-day food records at several intervals during weight maintenance. To complete 3-day food records, each participant recorded all food and beverages consumed during two weekdays and one weekend day. Participants were instructed to include as much detail as possible about all food and beverage items. A trained staff member reviewed and entered each dietary record and data were analyzed using the Nutrition Data System for Research (version 4.05_33).

#### Blood Collection

Overnight 12 hr fasting blood samples were collected at baseline, and after three, six, and twelve months. All blood samples were collected into 10 ml tubes containing EDTA using standard venipuncture methods by a trained phlebotomist. All samples were immediately separated by centrifugation for 15 min at 2000 g. The separated plasma was transferred to cryogenic vials and stored at -70°C for later analysis.

#### Lipid Subclasses

Fasting plasma samples were analyzed by LipoScience (LipoScience Inc, Raleigh, NC) using nuclear magnetic resonance spectroscopy (NMR) as described previously [18,19]. In brief, NMR identifies different lipoprotein subclasses based upon the idea that each lipoprotein particle emits a NMR signal that has a distinct frequency and shape. The intensities of these signals are proportional to the lipid mass concentration of that lipoprotein particle.

### Statistical Analysis

The level of significance was set at *p *< 0.05 for all statistical tests. The statistical software package PC-SAS (version 9.1, SAS Institute, Inc., Cary, NC) was used for tests of statistical significance. Independent T-tests were used to determine differences between groups at the beginning of weight loss and beginning of weight maintenance. Mixed models were utilized to determine group differences, within group changes across time, and/or group*time interactions for anthropometrics, energy intake from 3-day food records, and lipid subclasses. Analysis of lipid subclasses for a group*time interaction were adjusted for the individual subclass and body weight at the beginning of weight maintenance. For analysis of 3-day food records, adjusted means are reported to account for missing records. The General Linear Model was utilized to determine within group changes in weekly self-reported carbohydrate and fat grams across weight maintenance. When there was a missing data point for carbohydrate or fat gram intake in a particular week, the missing data was imputed by taking the average carbohydrate or fat gram from the previous month.

## Results

In the RC group, 21 individuals completed all testing and assessments (12 women and 9 men) and in the RF group 14 individuals completed all testing and assessments (11 women and 3 men). Participants were primarily Caucasian (94%). There were no significant differences at baseline between the RC group and RF group for body weight, BMI, or age (Table 1).

**Table 1:** Baseline characteristics by group.

	Reduced Carbohydrate	Reduced Fat	*F*	*p*
**N**	21	14		
**Weight (kg)**	110.3 ± 18.8	112.3 ± 17.6	0.10	0.75
**BMI (kg/m^**2**^)**	38.5 ± 4.5	40.3 ± 4.5	1.41	0.25
**Age (y)**	50.9 ± 8.9	45.6 ± 9.5	2.87	0.10

Mean ± SD.

Weight loss during the VLED was significant. Participants assigned to the RC diet lost ~20% of their body weight and the participants in the RF group lost ~19% of their body weight. Change in weight during the VLED was not different between groups (*p *> 0.05). Number of shakes during the VLED for all participants was 34.8 ± 2.8 equaling approximately 2092 kJ per day.

During weight maintenance, the RC group non-significantly increased body weight by ~2.8%. Likewise, the RF group showed a non-significant change of ~3.0% in body weight across weight maintenance; however, change in body weight across the study was not different between groups (*p *> 0.05). Similar trends were observed for BMI.

**Figure 1:**
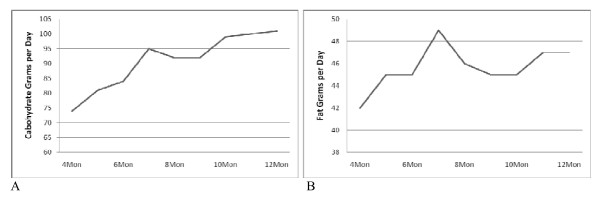
**Daily carbohydrate and fat grams for the reduced carbohydrate and reduced fat groups during weight maintenance**. **A**: Trend for increase in self-reported carbohydrate grams in the RC group was significant across weight maintenance (*F *= 9.36, *p *< 0.0001). **B**: Trend for increase in self-reported fat grams in the RF group was not significant across weight maintenance (*F *= 1.54, *p *= 0.152).

The RC group averaged approximately 25% of energy consumed as dietary carbohydrate while the RF group averaged approximately 28% of energy consumed as dietary fat during weight maintenance (Table 2). As expected, there was a significant difference between the RC and RF groups for carbohydrate and fat intake during weight maintenance (Table 2). There also was a significant difference in energy intake (kJ) between groups. Food records revealed that carbohydrate consumption increased in the RC group during weight maintenance and fat consumption did not statistically change in the RF group during weight maintenance. Self-reported carbohydrate grams for the RC group averaged 88.1 ± 32.7 throughout weight maintenance. However, there was a statistically significant increase (~36%) in the number of self-reported carbohydrate grams consumed from the beginning of weight maintenance to the conclusion of the study (*p *< 0.0001) (Figure 1A). Self-reported fat grams for the RF group averaged 44.3 ± 20.3 throughout weight maintenance and did not significantly increase across the duration of weight maintenance (*p *= 0.152) (Figure 1B).

**Table 2:** Macronutrient composition by group during weight maintenance.

	4-Months	6-Months	12-Months
**Reduced Carb (n = 21)**			
Energy Intake (kJ)	5415 ± 411	6524 ± 411	6774 ± 458*^†^
Carbohdyrates (g)	76 ± 9	101 ± 9	104 ± 10*^†^
Fat (g)	70 ± 7	83 ± 7	90 ± 8*^†^
Protein (g)	90 ± 8	100 ± 8	95 ± 9

**Reduced Fat (n = 14)**			
Energy Intake (kJ)	7048 ± 620	7457 ± 620	6500 ± 771
Carbohdyrates (g)	224 ± 18	231 ± 18	207 ± 23
Fat (g)	52 ± 7	58 ± 7	52 ± 9
Protein (g)	87 ± 7	89 ± 7	76 ± 9

Mean ± SE. Means are adjusted means.

*Indicates a significant difference within the Reduced Carbohydrate group over time. Changes were not significant over time for the Reduced Fat group.

^†^Indicates a significant difference from the Reduced Fat group.

During the weight maintenance period, the RC group showed a significant increase in mean LDL size, large LDL, total HDL, large HDL, small HDL particle concentration, mean VLDL size, and large VLDL (Table 3). On the other hand, the RF group showed a significant increase in total HDL, large HDL, small HDL, total VLDL, large VLDL, medium VLDL, and small VLDL particle concentration during the weight maintenance period. Tests for a group*time interaction revealed a significant interaction for large VLDL and medium VLDL, *p *= 0.044 and *p *= 0.049, respectively (Table 3).

**Table 3:** Lipids subclasses across weight maintenance.

Reduced Carbohydrate					Reduced Fat				
	**3-mon**	**6-mon**	**12-mon**	**Difference 12-3 mon**	**3-mon**	**6-mon**	**12-mon**	**Difference 12-3 mon**	***p*^†^**
	
**LDL**									
									
Mean LDL Size (nm)	20.9 ± 0.7^a^	21.5 ± 0.8^b^	21.2 ± 0.7^b^	0.03*	20.9 ± 0.4	21.0 ± 0.7	21.0 ± 0.8	0.1	0.228
Total LDL	1076 ± 228	1131 ± 328	1189 ± 340	113	1176 ± 333	1261 ± 481	1309 ± 494	133	0.945
Large LDL	372 ± 178^a^	544 ± 260^b^	486 ± 202^b^	114*	373 ± 120	423 ± 139	416 ± 185	43	0.131
Small LDL	663 ± 196	547 ± 382	671 ± 364	8	737 ± 260	782 ± 480	814 ± 564	77	0.347
Medium-Small LDL	133 ± 37	111 ± 78	139 ± 79	6	152 ± 52	162 ± 99	177 ± 123	25	0.407
Very-Small LDL	530 ± 163	436 ± 304	532 ± 287	2	585 ± 208	621 ± 382	637 ± 441	52	0.334
**HDL**									
									
Mean HDL Size (nm)	9.2 ± 0.4	9.3 ± 0.4	9.15 ± 0.4	-0.05	9.1 ± 0.3	9.0 ± 0.4	9.1 ± 0.4	0	0.282
Total HDL	25.7 ± 4.3^a^	33.0 ± 5.9^b^	36.3 ± 5.7^c^	10.6*	27.6 ± 3.6^a^	34.7 ± 6.0^b^	37.4 ± 4.6^c^	9.8*	0.845
Large HDL	5.9 ± 1.9^a^	8.2 ± 3.0^b^	8.8 ± 3.3^b^	2.9*	6.1 ± 2.1^a^	7.6 ± 3.4^b^	8.6 ± 4.0^b^	2.5*	0.530
Medium HDL	3.6 ± 2.6	2.5 ± 3.2	2.7 ± 2.8	-0.9	4.0 ± 2.5	4.9 ± 3.8	4.8 ± 3.5	0.8	0.112
Small HDL	16.2 ± 4.3^a^	22.3 ± 4.9^b^	24.8 ± 5.0^c^	8.6*	17.5 ± 3.9^a^	22.2 ± 6.5^b^	24.0 ± 5.2^b^	6.5*	0.441
**VLDL**									
									
Mean VLDL Size (nm)	43.1 ± 3.5^a^	44.6 ± 5.1^a^	48.4 ± 9.9^b^	5.3*	43.8 ± 5.6	47.3 ± 7.4	47.7 ± 11.8	3.9	0.445
Total VLDL	44.3 ± 26.5	49.4 ± 28.5	50.5 ± 29.8	6.2	56.2 ± 30.0^a^	77.1 ± 32.2^b^	76.0 ± 30.1^b^	19.8*	0.194
Large VLDL	0.7 ± 0.8^a^	0.6 ± 0.8^a^	1.5 ± 1.9^b^	0.8*	1.7 ± 2.5^a^	3.7 ± 3.9^b^	3.5 ± 4.9^b^	1.8*	**0.044**
Medium VLDL	13.3 ± 11.5	11.7 ± 10.0	15.5 ± 13.3	2.2	17.5 ± 13.5^a^	26.9 ± 13.5^b^	25.6 ± 16.5^b^	8.1*	**0.049**
Small VLDL	30.3 ± 16.8	37.1 ± 21.3	33.5 ± 18.8	3.2	37.0 ± 17.4^a^	46.5 ± 17.4^b^	46.9 ± 16.9^b^	9.9*	0.590

Mean ± SD. All units are nmol/L unless otherwise noted above.

*Significant change within group. Different subscripts indicate where within group changes occurred.

*p*^†^- Group*time interaction with control for individual subclass and body weight at the beginning of maintenance.

## Discussion

This study is unique as the RC and RF diets were consumed for weight maintenance rather than weight loss thereby reducing the effect of body weight change on the lipid outcomes measured. Taken as a whole, lipid subclass changes were similar between dietary groups across the duration of the study; however, a RC diet significantly attenuated the increase in some VLDL outcomes compared to the RF diet.

While the RF diet in the present study was consistent with the standard definition of a low fat diet (<30%), carbohydrate consumption in the RC group tended to be higher than some other studies in the literature. Accordingly, previous low carbohydrate and low fat studies have found different results in lipid subclasses compared to the present study [9,11,13]. It is possible that a moderate reduction in carbohydrate intake, as observed in the present study, is insufficient to produce more significant changes in lipid subclasses compared to a standard low fat diet when body weight is controlled. However, in a study by Seshadri et al., a carbohydrate content of ≤ 30 grams per day was prescribed for the low carbohydrate group but actual reported carbohydrate intake was 32 ± 20% (~113 g of carbohydrate per day) at the 6-month follow-up period [10]. It is noteworthy that Seshadri et al. reported somewhat similar lipid subclass trends to the present study, for example, a significant difference in change in large VLDL and lack of a significant difference in change in most other lipid subclasses between the low carbohydrate and low fat groups [10]. Overall, this begs the question of whether or not a carbohydrate consumption threshold exists to produce lipid subclass changes, beyond VLDL, that differ from those of a low fat diet.

Further, it is noteworthy that in the present study dietary carbohydrate consumption significantly increased across weight maintenance in the RC group (↑ ~36%) while dietary fat consumption did not statistically change in the RF group. Given the gradual and significant increase in carbohydrate content over time in the RC group, it is possible that lipid subclass changes would have been different had the RC group not increased their daily carbohydrate consumption; however, this is somewhat speculative.

While this study has the strength of being one of the first to compare a RC and RF diet for an extended period of weight maintenance (9 months) there also are limitations. Specifically, sample size was small. As a result, these data from may be most appropriately considered as pilot data for future studies. In addition, this study was quasi-experimental. This study design introduces the possibility that other factors or biases existed and influenced the results of the study.

In summary, this study suggests that following three months of significant weight loss on a VLED, a weight maintenance RC and RF diet show similar changes for lipid subclasses; however, medium VLDL and large VLDL did not increase to the same extent on the RC diet as the RF diet. A larger sample and stricter adherence to the RC diet may have altered the results of the study and may have yielded results that better differentiate between these diets for lipid subclass outcomes and potentially for CHD risk; this deserves further investigation.

## List of Abbreviations

RC: Reduced carbohydrate; RF: Reduced fat; LDL: Low-density lipoprotein; HDL: High-density lipoprotein; VLDL: Very low-density lipoprotein; CHD: Coronary heart disease; VLED: Very low-energy diet; NMR: Nuclear magnetic resonance spectroscopy.

## Competing interests

Dr. Mary C. Vernon received honoraria as a consultant for Mrs. Veronica Atkins, Chairperson of the Board of Directors for the Robert C. Atkins Foundation.

## Authors' contributions

JD conceived the study design with input from JL, BS, MV, and EW. JL coordinated and conducted the overall study with input from BS, EW, MV, and JD. MV provided medical oversight of all subjects. Data were analyzed by JL and BS. The manuscript was prepared by JL, BS, JD, and all authors contributed to the editing and input of the final manuscript.
